# Factors Impacting Survival After Transarterial Radioembolization in Patients with Unresectable Intrahepatic Cholangiocarcinoma: A Combined Analysis of the Prospective CIRT Studies

**DOI:** 10.1007/s00270-023-03657-x

**Published:** 2024-02-06

**Authors:** Peter Reimer, Valérie Vilgrain, Dirk Arnold, Tugsan Balli, Rita Golfieri, Romaric Loffroy, Cristina Mosconi, Maxime Ronot, Christian Sengel, Niklaus Schaefer, Geert Maleux, Graham Munneke, Bora Peynircioglu, Bruno Sangro, Nathalie Kaufmann, Maria Urdaniz, Helena Pereira, Niels de Jong, Thomas Helmberger

**Affiliations:** 1 Städtisches Klinikum Karlsruhe, Institute for Diagnostic and Interventional Radiology, Academic Teaching Hospital the University of Freiburg, Moltkestraße 90, 76133 Karlsruhe, Germany; 2 Université Paris Cité, CRI, INSERM, 1149 Paris, France; 3 Department of Radiology, Hôpital Beaujon APHP Nord, Clichy, France; 4 Oncology and Hematology, Asklepios Tumorzentrum Hamburg, AK Altona, Paul-Ehrlich-Str. 1, 22763 Hamburg, Germany; 5 Radiology Department, Çukurova University, Balcalı Hospital, 01330 Adana, Turkey; 6 Department of Radiology, IRCCS Azienda Ospedaliero-Universitaria Di Bologna, Bologna, Italy; 7 Department of Vascular and Interventional Radiology, Image-Guided Therapy Center, CHU Dijon Bourgogne, François-Mitterrand University Hospital, 14 Rue Gaffarel, 21000 Dijon, France; 8 Interventional Radiology, Centre Hospitalier Universitaire de Grenoble, Boulevard de La Chantourne, 38100 Grenoble, France; 9 Service de Médecine Nucléaire Et Imagerie Moléculaire, CHUV, Centre Hospitalier Universitaire Vaudois, Rue du Bugnon 46, CH–1011 Lausanne, Switzerland; 10 Radiology, Universitair Ziekenhuis Leuven, Herestraat 49, 3000 Leuven, Belgium; 11 Interventional Oncology, University College London Hospitals NHS Foundation Trust, 250 Euston Road, London, NW1 2PG UK; 12 Department of Radiology, School of Medicine, Hacettepe University, Sihhiye Campus, 06100 Ankara, Turkey; 13 Liver Unit and HPB Oncology Area, Clínica Universidad de Navarra and CIBEREHD, Avda. Pio XII 36, 31008 Pamplona, Spain; 14 Clinical Research Department, Cardiovascular and Interventional Radiological Society of Europe, Neutorgasse 9, 1010 Vienna, Austria; 15 Assistance Publique-Hôpitaux de Paris, Hôpital Européen Georges-Pompidou, Unité de Recherche Clinique, Paris, France; 16 INSERM, Centre d’Investigation Clinique 1418 (CIC1418), Paris, France; 17 Department of Radiology, Neuroradiology and Minimal-Invasive Therapy, Klinikum Bogenhausen, Englschalkinger Str. 77, 81925 Munich, Germany

**Keywords:** Intrahepatic cholangiocarcinoma, Observational, Transarterial radioembolization, Selective internal radiation therapy, Liver, Registries

## Abstract

**Purpose:**

Transarterial radioembolization (TARE) with Yttrium-90 resin microspheres is a treatment option for patients with intrahepatic cholangiocarcinoma (ICC). However, optimising the timing of TARE in relation to systemic therapies and patient selection remains challenging. We report here on the effectiveness, safety, and prognostic factors associated with TARE for ICC in a combined analysis of the prospective observational CIRT studies (NCT02305459 and NCT03256994).

**Methods:**

A combined analysis of 174 unresectable ICC patients enrolled between 2015 and 2020 was performed. Patient characteristics and treatment-related data were collected at baseline; adverse events and time-to-event data (overall survival [OS], progression-free survival [PFS] and hepatic PFS) were collected at every follow-up visit. Log-rank tests and a multivariable Cox proportional hazard model were used to identify prognostic factors.

**Results:**

Patients receiving a first-line strategy of TARE in addition to any systemic treatment had a median OS and PFS of 32.5 months and 11.3 months. Patients selected for first-line TARE alone showed a median OS and PFS of 16.2 months and 7.4 months, whereas TARE as 2nd or further treatment-line resulted in a median OS and PFS of 12 and 9.3 months (p = 0.0028), and 5.1 and 3.5 months (p = 0.0012), respectively. Partition model dosimetry was an independent predictor for better OS (HR 0.59 [95% CI 0.37–0.94], p = 0.0259). No extrahepatic disease, no ascites, and < 6.1 months from diagnosis to treatment were independent predictors for longer PFS.

**Conclusion:**

This combined analysis indicates that in unresectable ICC, TARE in combination with any systemic treatment is a promising treatment option.

**Level of evidence**: level 3, Prospective observational

**Graphic Abstract:**

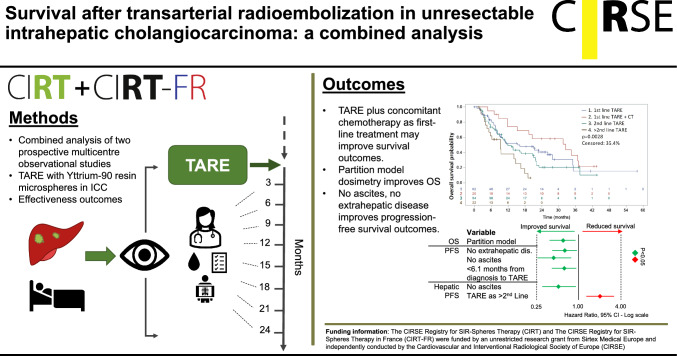

**Supplementary Information:**

The online version contains supplementary material available at 10.1007/s00270-023-03657-x.

## Introduction

Intrahepatic cholangiocarcinoma (ICC) is the second most common primary liver malignancy after hepatocellular carcinoma (HCC). The incidence of ICC and ICC-related deaths are increasing, especially in Western countries, where tumours are associated with a dismal prognosis and short overall survival (OS) [1–4]. Complete surgical resection represents the only curative intent therapy [5–7]; however, 70% to 85% of ICC patients present with advanced disease where resection is no longer a treatment option [8–11].

First-line systemic therapy in patients with non-resectable tumours included gemcitabine or gemcitabine and cisplatin-based regimens, resulting in a median overall survival (OS) of 8.1 months and 11.7 months, respectively [12, 13] as a standard of care for many years. More recently, the administration of immune checkpoint inhibitors durvalumab or pembrolizumab in addition to a standard first-line combination chemotherapy resulted in a median OS likelihood of 12.8 and 12.7 months, respectively [14, 15]. For further line treatment, a European trial showed improvement in OS with the FOLFOX regimen compared to active symptom control (hazard ratio [HR] 0.69 [95% confidence interval [CI] 0.50–0.97, p = 0.031) [16], while a phase 3 trial in South Korea showed improved progression-free survival (PFS) when adding liposomal irinotecan to fluorouracil and leucovorin compared to fluorouracil and leucovorin alone (HR 0.56, 95% CI 0.39–0.81, p = 0.0019) [17]. However, resistance to systemic treatment is a known limitation in managing patients with ICC [18, 19].

Transarterial radioembolization (TARE) is an interventional therapeutic procedure that involves the targeted delivery of high doses of radiation to liver tumours via the hepatic artery. While robust evidence on the effectiveness of TARE in ICC is lacking, small population studies have suggested that TARE in the first-line palliative setting may provide additional benefits to the patients in light of other available systemic therapies [20]. Current guidelines indicate that patients with ICC may also benefit from receiving TARE as a second-line treatment after systemic therapy [5, 6].

The Cardiovascular and Interventional Radiological Society of Europe (CIRSE) initiated two prospective observational studies on the clinical application and outcomes of TARE with Yttrium-90 (Y90) resin microspheres (SIR-Spheres® Y90 resin microspheres, Sirtex Medical Pty Limited; St. Leonards, NSW, Australia): under the acronym CIRSE Registry for SIR-Spheres Therapy (CIRT), a Europe-wide cohort (NCT02305459) and a France-only cohort (NCT03256994) were collected. The present analysis combined the ICC cohorts collected in these CIRT studies to evaluate effectiveness outcomes after TARE in ICC and identify clinical characteristics as (potential) prognostic factors for effectiveness outcomes, to inform the optimal patient selection and treatment strategies.

## Materials and Methods

### Study Design

A pooled cohort of ICC patients from the Europe-wide and from the France-only studies were analysed. The CIRT studies are prospective, single-device, multi-centre observational studies with primary and metastatic hepatic malignancies treated with TARE using Y90 resin microspheres as the standard of care. The methodology of the CIRT study concept was published by Helmberger et al. [21]. For more insights on both cohorts, please refer to previously published papers [22–25].

In the European cohort, sites were invited to participate if they had a history of at least forty TARE cases, including ten cases within the twelve months prior to invitation. In the French cohort, all sites where TARE was performed were invited to participate regardless of prior experience with the treatment. Patient recruitment took place between January 2015 and December 2017, and between August 2017 and August 2020 in the CIRT and French CIRT studies, respectively. Follow-up data were collected until December 2019 in CIRT and until July 2022 in the French CIRT.

Data was collected using a customised electronic data capturing system and electronic case report form developed by ConexSys Inc. (Lincoln, RI, United States) and hosted on a local secure server in Vienna, Austria, maintained by ITEA (Vienna, Austria). Statistical analyses were performed in SAS 9.4 (SAS Institute, Cary, NC, USA) and RStudio under R4.0.0 (R Foundation, Vienna, Austria).

### Patient Selection

Patients included in the analysis were adults with histologically confirmed ICC and scheduled to receive TARE with Y90 resin microspheres. There were no specific exclusion criteria. All included patients signed an informed consent form. Procedures were performed in accordance with the ethical standards of the institutional and/or national research committee and with the 1975 Helsinki Declaration and its later amendments or comparable ethical standards.

Participating sites were recommended to follow up with the patient every three months after the first TARE treatment. Due to the observational nature, actual follow-up intervals were left at the discretion of the investigators.

### Assessments and Definitions

At the time of the first treatment, patient demographics, baseline data and treatment-related data were collected. Post-TARE treatments, safety data and time-to-event data were collected at every follow-up. Time-to-event was defined from the date of the first TARE treatment until the event date. Safety outcomes are described according to the Common Terminology Criteria for Adverse Events, version 4.03. Clinical parameters were disease status, tumour burden, procedures before and after TARE and dose methodology, as well as relevant blood markers including albumin, bilirubin, liver transaminases, International Normalised Ratio (INR) and Albumin-Bilirubin (ALBI) Grade (see Supplement 1 for the ALBI formula). Concomitant therapy was defined as the start of any systemic treatment with 56 days (8 weeks) before or after TARE.

### Statistical Analysis

The datasets from both cohorts were combined and analysed. Since the case report forms and study proceedings were the same, no additional data manipulation was necessary. Data are presented as mean ± standard deviation or median (interquartile range [IQR]) for continuous variables and number (%) for categorical variables. Patients that died during the study were categorised as having progression for the PFS and hepatic PFS (hPFS) analysis.

The median OS, PFS and hPFS time were calculated with the associated 95% confidence interval using the Kaplan–Meier method and the median follow-up period was calculated using the reverse Kaplan–Meier method. A p-value of > 0.05 was considered statistically significant.

Multivariable survival analysis for OS, PFS and hPFS was performed using a Cox proportional-hazards model. The selection of variables was determined following a univariable analysis and a subsequent stepwise variable selection procedure with a significance level of 0.2 when deciding to enter a predictor into the stepwise model. The model with the lowest Akaike information criterion value was considered the final model. All available data were used, and no imputations of missing data were made. Missing data is indicated in the summary tables.

## Results

### Patient Demographics

One hundred seventy-four patients with ICC from 26 centres in eight European countries were included in this study, 120 patients from the European cohort and 54 from the French cohort (see Supplement 2). The median age was 64 (IQR 57–72), and 97/174 (55.7%) were male. The median time from diagnosis until first TARE was 6.1 months. Patients were in relatively good condition with Eastern Cooperative Oncology Group (ECOG) status mostly 0 (97, 55.7%) or 1 (61, 35.1%), and no extrahepatic disease in 124 (71.3%) of the patients (Table 1). Ascites and cirrhosis were observed in 13 (7.5%) and 21 (12.1%) of the patients, respectively. ALBI grade 1 was observed in 72 (41.4%) patients, grade 2 in 67 (38.5%) patients, and grade 3 in 1 (0.6%) patient. Baseline data from both cohorts separately are presented in Supplement 2.

**Table 1:** Baseline characteristics

Category	Subcategory	ICC (n = 174)		
Age	n	174 (100)		
	Median	64		
	IQR	57–72		
	Range	29–88		
Sex	n	174 (100)		
	Female	73 (42)		
	Male	97 (55.7)		
	Unknown	4 (2.3)		
Time since diagnosis (months)	n	172 (98.9)		
	Median	6.1		
	IQR	2.2–14.5		
ECOG status	n	174 (100)		
	0-Fully Active	97 (55.7)		
	1-Restricted	61 (35.1)		
	2 or higher	12 (6.9)		
	Missing	4 (2.3)		
Extrahepatic disease prior to treatment	n	174 (100)		
	No	124 (71.3)		
	Yes	50 (28.7)		
Ascites	n	174 (100)		
	No	161 (92.5)		
	Yes	13 (7.5)		
Cirrhosis	n	174 (100)		
	No	153 (87.9)		
	Yes	21 (12.1)		
Location of liver tumours	n	174 (100)		
	Bilobar	86 (49.4)		
	Left only	27 (15.5%)		
	Right only	61 (35.1%)		
Number of liver tumours	n	174 (100)		
	1	80 (46)		
	2–5	35 (29.2)		
	6–9	11 (6.3)		
	10 or more	12 (6.9)		
	Uncountable	36 (20.7)		
Methodology for determining the dose	n	174 (100)		
	BSA or modified BSA	87 (50)		
	Partition model	87 (50)		
Albumin (g/dL)	n	141 (81)		
	Median (IQR)	3.8 (3.4–4.2)		
Bilirubin (µmol/L)	n	172 (98.9)		
	Median (IQR)	10 (7.2–14)		
ALBI score	n	140 (80.5)		
	Grade 1	72 (41.4)		
	Grade 2	67 (38.5)		
	Grade 3	1 (0.6)		
INR	n	134 (77)		
	Median (IQR)	1.1 (1.0, 1.1)		
	≤ 1	51 (29.3%)		
	> 1	83 (47.7)		
		**Whole liver**	**Left lobe**	**Right lobe**
Percentage of tumour invasion in the liver	n	123	43	45
	< 10%	46 (26.4)	16 (9.2)	22 (12.6)
	10–20%	37 (21.3)	15 (8.6)	10 (5.7)
	> 20%	40 (23)	12 (6.9)	13 (7.5)
Tumour burden	n	170 (97.7)		
	< 10%	60 (35.3)		
	10–20%	58 (34.1)		
	> 20%	52 (31.6)		
Prescribed activity (Giga-becquerel)	n	91	83	83
	Median	1.2	0.6	1.1
	IQR	0.9–1.6	0–1.0	0.7–1.4
Liver treatment target	n	174 (100)		
	Whole liver (single catheter)	13 (7.5)		
	Whole liver (split administration, single session)	32 (18.4)		
	Whole liver (sequential lobar, two sessions)	17 (9.8)		
	Right lobe	62 (35.6)		
	Left lobe	34 (19.5)		
	Segmental	16 (9.2)		
Delivered activity within 90% of prescribed (technical success)	n	174 (100)		
	No	4 (2.3)		
	Yes	170 (97.7)		

N (%)

*ALBI* albumin-bilirubin, *BSA* body surface area, *ECOG* Eastern Cooperative Oncology Group, *ICC* intrahepatic cholangiocarcinoma, *INR* international normalised ratio, *IQR* interquartile range

Categories where percentages (%) do not add up to 100 are due to missing information

### Treatment Planning and Application

Bilobar tumours were found in 86 (49.4%) patients, and patients had one tumour (80, 46%), two to five tumours (35, 29.2%), six to nine tumours (11, 6.3%), ten or more tumours (12, 6.9%) or an uncountable number of tumours (36, 20.7%) (more details in Table 1). Tumour burden was < 10% (60, 35.3%), 10–20% (58, 34.1%) or > 20% (52, 31.6%). The prescribed activity was calculated using partition model dosimetry (50%) or body surface area (BSA) and modified BSA (50%). The median prescribed activity was 1.2 Giga-becquerel (GBq) (IQR 0.9–1.6) for whole liver treatments, 1.1 GBq (IQR 0.7–1.4) for right lobe treatments and 0.6 GBq (IQR 0.0–1.0) for left lobe treatments. The delivered activity was within 90% of the prescribed activity (i.e., technical success) in 170 (97.7%) cases.

The investigator-reported intention of TARE was primarily palliative (128, 73.6%) or downsizing (33, 19%) to potential curative treatment without pre-defined subsequent treatment (Table 2). Before TARE, 49 (28.2%) patients received locoregional treatments, mostly surgical resection (37, 75.5%) as opposed to transplantation. Sixty-two patients (35.6%) received TARE as monotherapy at first line, 20 (11.5%) received first-line TARE with concomitant systemic treatment, 54 patients (31%) had already received one line of systemic treatment, and 22 (12.6%) had received two or more lines of systemic treatment. After TARE, patients underwent additional locoregional treatments (16.7%) and/or systemic therapy sessions (48.3%).

**Table 2:** Treatments before and after TARE

Category	Subcategory	ICC (n = 174)
Intention of treatment^a^	n	174 (100)
	Palliative	128 (73.6)
	Downsizing	33 (19)
	Bridge to ablation	8 (4.6)
	Bridge to liver surgery	3 (1.7)
	Bridge to liver transplant	2 (1.1)
Position of TARE in the continuum of care	n	158 (90.8)
	First-line TARE	62 (35.6)
	First-line TARE plus concomitant systemic therapy^c^	20 (11.5)
	Second line TARE	54 (31)
	> 2nd line TARE	22 (12.6)
Hepatic procedures prior to TARE	n	174 (100)
	Yes	49 (28.2)
	No	125 (71.8)
Type of hepatic procedures^b^	n	49 (100)
	Surgical (any)	37 (75.5)
	Ablation (any)	8 (16.3)
	TACE (any)	2 (4.1)
	Other embolotherapies (any)	1 (2)
	Abdominal radiotherapy (any)	9 (18.4)
Systemic therapy after TARE	n	174 (100)
	Yes	84 (48.3)
	No^c^	90 (51.7)
Number of systemic therapy lines after TARE	n	83 (98.8)
	1 Line	37 (44)
	2–5 Lines	31 (36.9)
	6 or more lines	15 (17.6)
Hepatic procedures after TARE	Yes	29 (16.7)
	No	145 (83.3)
Type of hepatic procedures after TARE^d^	n	29 (100)
	Surgical (any)	12 (41.4)
	Ablation (any)	5 (17.2)
	TACE (any)	1 (3.4)
	Other embolotherapies (any)	4 (13.8)
	Abdominal radiotherapy (any)	12 (41.4)
	Not reported	3 (10.3)

N (%)

^a^Intention of TARE is for first treatment

^b^Patients can have multiple prior and post-TARE hepatic procedures

^c^Concomitant therapy is defined as any systemic therapy that starts within 56 days before or after TARE

^d^No systemic therapy after TARE includes patients that were lost to follow-up or deceased before the first follow-up could be included (12 [10] for CIRT and 2 [3.7] for CIRT-FR)

*ICC* intrahepatic cholangiocarcinoma, *TACE* transarterial chemoembolization, *TARE* transarterial radioembolization

Categories where percentages (%) do not add up to 100 are due to missing information

### Effectiveness

**Fig. 1:**
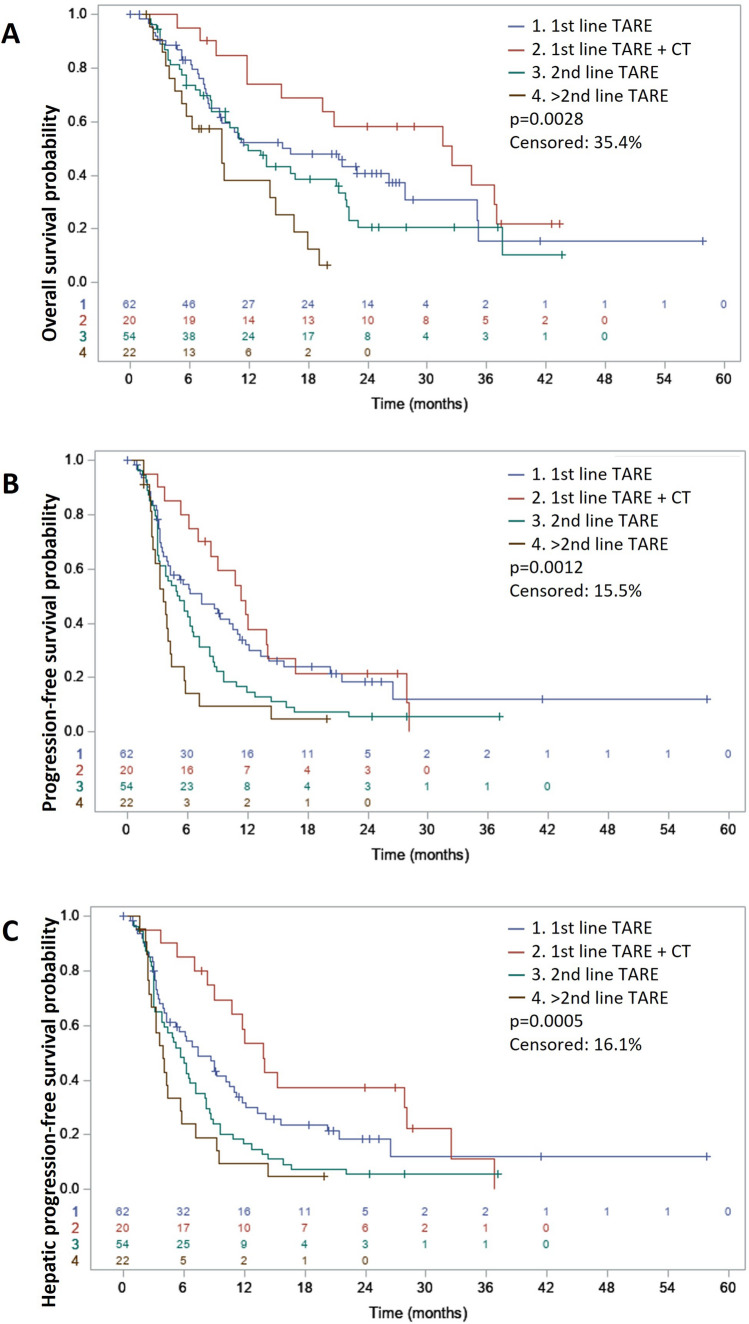
Kaplan–Meier plot showing differences in (**A**) overall survival, (**B**) progression-free survival and (**C**) hepatic progression-free survival for 1st line TARE, 1st line TARE + concomitant therapy (CT), 2nd line TARE, and > 2nd line TARE. Levels of significance: p < 0.05 (Log-rank test)

After a median follow-up of 26.2 months (per reverse Kaplan–Meier, 95% CI 24.4–28.5), 114 (65.5%) patients died, and 101 (58%) showed hepatic progression. The median OS for the entire cohort was 15.3 months (95% CI 11.2–19.1). Patients that received first-line TARE plus concomitant systemic treatment (20, 11.5%) had the longest median OS: 32.5 months (95% CI 11.8–37.0), patients receiving first-line TARE alone (62, 35.6%) had a median OS of 16.2 months (95% CI 9.0–27.7), patients receiving TARE as second line (with or without concomitant chemotherapy, 54, 31%) had a median OS of 12.0 months (95% CI 8.2–20.8), and at further lines (22, 12.6%) the median OS was 9.3 months (95% CI 4.5–14.7), p = 0.0028 (shown in Fig. 1A). A comparison between baseline characteristics between patients receiving first-line TARE plus concomitant systemic treatment and first-line TARE alone reveals no statistically significant differences between the cohorts (Supplement 3). The time from diagnosis to TARE per treatment line is reported in Supplement 4. Univariable analysis (Table 3) also showed favourable OS outcomes for patients with no extrahepatic disease or cirrhosis, partition model dosimetry and additional (locoregional and systemic) treatments after TARE. In the multivariable analysis (Table 4), partition model dosimetry (HR 0.59 [95% CI 0.37–0.94], p = 0.0259) was the only statistically significant independent prognostic factor for OS.

**Table 3:** Prognostic factors for overall survival (OS), progression-free survival (PFS), and hepatic PFS (hPFS)

Category		Median (95% CI)	p-value	HR (95% CI)	p-value HR
**Overall survival**					
Position of TARE in the continuum of care	1st line TARE	16.2 (9.0–27.2)	0.0028		
	1st line TARE plus CT	32.5 (11.8–27.0)		0.64 (0.34–1.22)	0.1730
	2nd line TARE	12.0 (8.2–20.8)		1.28 (0.82–2.02)	0.2817
	> 2nd line TARE	9.3 (4.5–14.7)		2.37 (1.31–4.29)	0.0042
Extrahepatic disease prior to TARE	No	18.4 (11.8–22.9)	0.0023	0.55 (0.37–0.81)	0.0026
	Yes	10.9 (6.2–14.7)			
Cirrhosis	No	16.6 (13.2–21.1)	0.0051	0.47 (0.28–0.81)	0.0062
	Yes	7.7. (3.8–10.0)			
Dose methodology	BSA/mBSA	12.0 (9.4–16.6)	0.0363	0.67 (0.46–0.98)	0.0379
	Partition model	17.1 (10.1–26.2)			
Locoregional treatments after TARE	No	11.8 (9.7–16.6)	0.0101	1.98 (1.17–3.37)	0.0116
	Yes	27.7 (16.2–37.0)			
Systemic therapy after TARE	No	9.3 (7.0–14.0)	0.0008	1.87 (1.29–2.71)	0.0010
	Yes	20.6 (14.7–26.2)			
Additional treatments after TARE	No	8.2 (5.7–11.2)	0.0000	2.36 (1.62–3.43)	0.0000
	Yes	20.8 (15.4–27.7)			
**Progression-free survival**					
Position of TARE in the continuum of care	1st line TARE	7.4 (3.9–11.0)	0.0012		
	1st line TARE plus CT	11.3 (6.1–14.0)		0.80 (0.46–1.40)	0.4405
	2nd line TARE	5.1 (3.1–6.4)		1.56 (1.04–2.33)	0.0300
	> 2nd line TARE	3.5 (2.5–4.3)		2.36 (1.39–4.04)	0.0016
Extrahepatic disease prior to TARE	No	6.9 (5.2–8.6)	0.0025	0.59 (0.42–0.83)	0.0028
	Yes	3.6 (3.0–6.0)			
Time from diagnosis to treatment	< 6.1	7.5 (5.3–10.2)	0.0054	1.59 (1.15–2.22)	0.0057
	> = 6.1	4.4 (3.8–6.2)			
Total tumour to liver (%)	< 10%	7.3 (4.4–12.0)	0.0602		
	10–20%	7.1 (4.4–9.3)		1.44 (0.89–2.34)	0.1414
	> 20%	4.4 (3.5–7.0)		1.74 (1.09–2.80)	0.0210
**Hepatic progression-free survival**					
Position of TARE in the continuum of care	1st line TARE	7.5 (4.3–11.0)	0.0005		
	1st line TARE plus CT	13.8 (8.3–28.1)		0.63 (0.35–1.12)	0.1140
	2nd line TARE	5.7 (3.8–7.2)		1.54 (1.03–2.30)	0.0337
	> 2nd line TARE	3.9 (2.6–5.7)		2.15 (1.27–3.67)	0.0047
Extrahepatic disease prior to TARE	No	7.4 (5.7–9.3)	0.0032	0.60 (0.42–0.84)	0.0035
	Yes	5.0 (3.1–6.4)			
Time from diagnosis to treatment	< 6.1	8.6 (6.1–10.7)	0.0021	1.68 (1.20–2.35)	0.0023
	> = 6.1	5.7 (4.0–7.1)			

Cox model, p-value 0.05. Only variables with a p < 0.05 are shown here. A complete overview of the outcomes of the univariable analyses for OS, PFS and hPFS can be found in **supplements 4–6**

Levels of significance: p < 0.05 (Log-rank test [Mantel–Haenszel version]). The following variables were considered: Gender; ECOG; Extrahepatic disease prior to treatment; Location of liver tumors; Ascites; Cirrhosis; Prior surgery; Dose methodology; Treatment intention; Prior locoregional treatments; Locoregional treatments after TARE; Systemic therapy after TARE; Additional treatments after TARE; Time from diagnosis to treatment (months); Total tumour to liver (%); Right tumour to liver (%); Left tumour to liver (%); ALBI grade and International Normalized Ratio

*ALBI* albumin-bilirubin, *BSA* body surface area, *CI* confidence interval, *CT* concomitant treatment, *ECOG* Eastern Cooperative Oncology Group, *ICC* intrahepatic cholangiocarcinoma, *HR* hazard ratio, *TARE* transarterial radioembolization

**Table 4:** Prognostic factors for overall survival (OS), progression-free survival (PFS), and hepatic PFS (hPFS)

Variable	Category	HR (95% CI)	p-value
**Overall survival**			
Extrahepatic disease prior to TARE	No	0.64 (0.40–1.02)	0.0592
Dose methodology	Partition model	0.59 (0.37–0.94)	**0.0259**
**Progression-free survival**			
Extrahepatic disease prior to TARE	No	0.64 (0.44–0.92)	**0.0177**
Ascites	No	0.45 (0.23–0.87)	**0.0180**
Locoregional treatments after TARE	No	1.52 (0.92–2.52)	0.1053
Time from diagnosis to treatment	< 6.1 months	0.67 (0.47–0.97)	**0.0350**
**Hepatic progression-free survival**			
Position of TARE in the continuum of care, compared to 1st line TARE	1st line TARE + CT	0.60 (0.34–1.08)	0.0880
	2nd line TARE	1.38 (0.90–2.11)	0.1350
	> 2nd line TARE	1.90 (1.10–3.28)	**0.0224**
Extrahepatic disease prior to TARE	No	0.69 (0.47–1.01)	0.0581
Ascites	No	0.51 (0.26–0.99)	**0.0468**

Cox proportional-hazards model, p-value 0.05

Levels of significance: p < 0.05 (Cox proportional-hazards model). The proportional hazard function of the Cox model was verified. 121 patients were included for overall survival; 156 patients were included for progression-free and hepatic progression-free survival

*CI* confidence interval, *CT* concomitant therapy, *HR* hazard ratio, *TARE* transarterial radioembolization

The median PFS was 6.0 months (95% CI 4.4–7.2), and the median hPFS was 6.4 months (95% CI 5.3–8.2). The position of TARE in the continuum of care (shown in Fig. 1B, C), no extrahepatic disease, and < 6.1 months from diagnosis until TARE predicted an improved PFS and hPFS, while < 20% tumour burden predicted an improved PFS (Table 3). In the multivariable analysis (Table 4), no extrahepatic disease, no ascites, and < 6.1 months from diagnosis to treatment were independent predictors for longer PFS (HR 0.64 [95% CI 0.44–0.92], p = 0.0177, HR 0.45 [95% CI 0.23–0.87], p = 0.0180, and HR 0.67 [0.47–0.97], p = 0.0350, respectively). For hPFS, independent predictors were no ascites (HR 0.51 [0.26–0.99], p = 0.0468) and TARE in the third line or beyond compared to first-line TARE (HR 1.90 [1.10–3.28], p = 0.0224). The complete univariable analyses for OS, PFS and hPFS can be found in Supplements 5–7.

### Safety

During the study, 89 patients (51.1%) experienced at least one adverse event (Table 5), primarily mild (grade 1–2) adverse events such as abdominal pain (19.5%), fatigue (30.5%) or nausea (17.8%). Rarely, gastrointestinal ulcerations (3.4%), gastritis (1.1%) or REILD (2.3%) were observed. Severe adverse events (grade 3 and 4) were found in 28 (16.1%) patients: abdominal pain 5 (2.9%), fatigue 5 (2.9%), gastrointestinal ulceration 1 (0.5%), gastritis 1 (0.5%), radiation cholecystitis 1 (0.5%), REILD 3 (1.7%), and other 22 (12.6%). Supplement 9 shows the safety outcomes from the European and French cohorts separately.

**Table 5:** Safety

Category	Subcategory	All grades	Grade 3–5
Patients with adverse events	n	174 (100)	174 (100)
	No. of patients with at least one adverse event	89 (51.1)	28 (16.1)
Adverse events (all)	Abdominal pain	34 (19.5)	5 (2.9)
	Fatigue	53 (30.5)	5 (2.9)
	Fever	9 (5.2)	0 (0)
	Nausea	31 (17.8)	0 (0)
	Vomiting	13 (7.5)	0 (0)
	Gastrointestinal ulceration	6 (3.4)	1 (0.5)
	Gastritis	2 (1.1)	1 (0.5)
	Radiation cholecystitis	1 (0.5)	1 (0.5)
	Radiation pancreatitis	0 (0)	1 (0.5)
	Radioembolization-induced liver disease	4 (2.3)	3 (1.7)
	Other	59 (33.9)	22 (12.6)

## Discussion

The present analysis results from the combined ICC cohorts of the prospective observational CIRT studies. Results indicate that patients receiving a combination of TARE with any systemic treatment as first-line treatment may have favourable OS, PFS and hPFS outcomes compared to TARE alone or to those receiving systemic therapy first. Moreover, further locoregional or systemic treatments after TARE are associated with favourable survival outcomes. Patients for whom dosimetry was determined using partition model dosimetry had longer OS outcomes than patients for whom dosimetry was determined by BSA or modified BSA. In terms of patient selection, patients without ascites and extrahepatic disease had better survival outcomes. Generally, safety results showed a good tolerability profile with 16.1% of the patients having reported any serious adverse events, 1.7% of these being REILD.

### TARE and Systemic Therapy

The position of TARE in the treatment pathway of patients with ICC is contended. Our data show that patients treated with TARE plus concomitant systemic treatment had the best OS, PFS, and hPFS outcomes, compared to TARE alone or TARE after one or more lines of systemic treatments. However, the fact that these results did not maintain in the multivariable analysis may point towards a difference in patient presentation or other factors unaccounted for, which would go beyond the scope of this paper to explore in full. The benefits of TARE in treatment-naïve patients have been highlighted by other studies—albeit with smaller populations [26–28]. In 2017, Cucchetti et al. performed a meta-regression study and concluded that treatment-naïve patients with mass‐forming ICC should be selected as the best candidates with the possibility of adding concomitant standard systemic therapy [29]. In the phase 2 clinical trial published by Edeline et al., the authors concluded that a TARE plus concomitant systemic treatment (given one day before or after TARE) bears significant antitumor activity when used as first-line treatment, achieving an OS of 22 months [30]. At the same time, outcomes from the prospective observational RESiN study suggest that patients that received one line of systemic therapy had better outcomes after TARE compared to systemic therapy-naïve patients (19.1 vs 10.6 months, p = 0.07) [31], while a retrospective multicentre analysis of 128 ICC patients by Schaarschmidt et al. did not report any differences in survival related to treatment lines (p = 0.15) but did report an improved overall survival at any stage of treatment from first-line to salvage treatment due to the addition of TARE [32].

Recent randomised controlled trials have shown that the addition of immune checkpoint inhibitors durvalumab or pembrolizumab to standard first-line combination chemotherapy resulted in an improvement of OS likelihood of 1.3 months and 1.7 months, respectively, and is now considered as the standard of care [14, 15]. In our prospective observational setting, the patients that received first-line TARE plus any concomitant systemic treatment (within 56 days of TARE) had a median OS of 32.5 months. Despite the study design and potential confounding factors, TARE in combination with systemic treatment could be a promising first-line treatment in patients with unresectable ICC. Further research into the molecular structures of ICC suggests that patients with fibroblast growth factor receptor (FGFR)-2 fusions respond better to second-line therapies [33, 34], potentially complicating the results of systemic treatments in ICC. In the meantime, the outcome of this prospectively collected real-world dataset provides further insight into the optimal place of TARE in the treatment pathway of patients with ICC.

### Prognostic Factors

The multivariable analysis identified partition model dosimetry as an independent prognostic factor for an improved OS. Evidence from the recent Phase 2 DOSISPHERE-01 trial showed that personalised dosimetry methods such as partition model dosimetry in glass microspheres could improve survival outcomes in patients with HCC, compared to standard dosimetry [35, 36], which was recently confirmed in resin microspheres by a large prospective observational cohort [37]. Based on the evidence in HCC, an international expert group recommended using partition model or voxel-based dosimetry for activity prescription in resin microspheres when either whole liver or selective, non-ablative or ablative TARE is planned, with a mean absorbed dose to non-tumoural liver of 40 Gy and minimum mean tumour-absorbed dose of 100–120 Gy [38]. Indeed, a small prospective study with 38 ICC patients suggested that for patients receiving resin microspheres, a mean tumour dose of ≥ 75 Gy or a maximum tumour dose of ≥ 150 Gy was associated with a median OS of 20.2 months compared to 6.5 months for those receiving less (p = 0.001 and 0.002, respectively) [39]. This prospective cohort confirms that real-world patients with ICC may benefit from personalised dosimetry compared to standard dose calculation models (BSA and modified BSA). Our study was not designed with detailed dosimetry-related outcomes in mind, and further research into this topic should be considered.

Several systematic reviews and meta-analyses on studies on TARE in ICC found similar OS results as this study but underlined the significant heterogeneity of treated patients in the retrieved studies [20, 40–42]. Our results emphasise the observations from previous studies that a local treatment such as TARE should not be the treatment of choice in ICC patients with extra-hepatic disease, ascites, or extensive tumour burden [27, 30, 43–50]. Other studies have identified prior treatments [47, 51] and tumour response [27, 45, 50, 52] as other significant prognostic factors. Köhler et al. found that the extent of liver disease to one or both liver lobes was associated with survival, irrespective of tumour volume (p = 0.041) [51].

### Limitations

Limitations of this prospective observational study are the existence of potentially critical confounding factors, which could not be controlled. The heterogeneity of the patient population reflects the real-life clinical practice in participating sites and, thus, its diversity in patient selection and clinical outcomes. We used a multivariable analysis to alleviate, to some degree, this heterogeneity but potential confounding factors not included in the analysis should be considered when interpreting the results. It is essential to consider that the patients in the European cohort were recruited between 2015 and 2017, while the patients in the French cohort were recruited between 2017 and 2019. Any potential changes in practice over time, for example changes in the delivery systems, imaging models allowing for more precise tumour targeting and better patient selection were not considered when analysing the results but should be considered when interpreting them.

The relatively high number of censored patients for the analysis of OS (60, 34.5%) and PFS (27, 15.5%) is comparable to other studies in oncology [53]. Investigators’ reports confirmed that TARE requires a comprehensive hospital infrastructure, leading to referrals from physicians who follow up with the patient after the treatment. Follow-up information was, in those cases, obtained by contacting the referring physician or, if this was not possible, the patient was considered as lost to follow-up. Moreover, interventional radiology departments did not always have the appropriate infrastructure to perform follow-ups consistently, contributing to the increased number of censored patients during follow-ups. Finally, central tumour response assessment was not done. Instead, response assessment was performed by the sites with various criteria (e.g., Response Evaluation Criteria in Solid Tumours (RECIST), modified RECIST or Positron Emission Tomography Response Criteria in Solid Tumours (PERCIST)) according to local habits and expertise of centres. This prevented us from including tumour response in the analysis.

## Conclusion

This analysis represents a large prospective cohort of patients with nonresectable ICC treated with TARE. Despite the limitations of a real-world study, the results suggest that TARE combined with concomitant systemic treatment could be considered as an early treatment modality in the treatment pathway of patients with liver-only ICC, also considering its low toxicity. Our findings suggest the need for more studies to account for further confounders and to be able to draw confident conclusions about these combination treatments.

### Supplementary Information

Below is the link to the electronic supplementary material.

Supplementary file1 (DOCX 90 KB)
