# Supplementary material for: Factors Impacting Survival After Transarterial Radioembolization in Patients with Unresectable Intrahepatic Cholangiocarcinoma: A Combined Analysis of the Prospective CIRT Studies
Source: Cardiovasc Intervent Radiol. 2024 Feb 6;47(3):310–24. doi: 10.1007/s00270-023-03657-x (PMC10920466; doi:10.1007/s00270-023-03657-x)
Supplement: Supplementary file 1 — Supplementary file1 (DOCX 90 KB) [file 270_2023_3657_MOESM1_ESM.docx]

Supplements to:

**Factors impacting survival after transarterial radioembolization in patients with intrahepatic cholangiocarcinoma: A combined analysis of the prospective CIRT studies**

Peter Reimer, Valérie Vilgrain, Dirk Arnold, Tugsan Balli, Rita Golfieri, Romaric Loffroy, Cristina Mosconi, Maxime Ronot, Christian Sengel, Niklaus Schaefer, Geert Maleux, Graham Munneke, Bora Peynircioglu, Bruno Sangro, Nathalie Kaufmann, María Urdaniz, Helena Pereira, Niels de Jong, Thomas Helmberger

**Contents**

[Supplement 1: ALBI formula 2](#_Toc148369731)

[Supplement 2: Baseline and treatment data from the European and French cohorts 3](#_Toc148369732)

[Supplement 3: A baseline comparison between patients receiving first-line TARE versus first-line TARE plus concomitant systemic therapy 6](#_Toc148369733)

[Supplement 4: Time from diagnosis to TARE treatment per treatment line 8](#_Toc148369734)

[Supplement 5: Univariable analysis for overall survival. 9](#_Toc148369735)

[Supplement 6: Univariable analysis for progression-free survival. 11](#_Toc148369736)

[Supplement 7: Univariable analysis for hepatic progression-free survival. 13](#_Toc148369737)

[Supplement 8: Safety data from the European and French cohorts 15](#_Toc148369738)

## **Supplement 1: ALBI formula**

ALBI score = (log^10^ bilirubin [µmol/L] × 0.66) + (albumin [g/L] × −0.0852), whereas ALBI score ≤ −2.60 is grade 1, > −2.60 to ≤ −1.39 is grade 2, and > −1.39 is grade 3.

## **Supplement 2: Baseline and treatment data from the European and French cohorts**

**Baseline characteristics**

| **Category** | **Subcategory** | **European cohort (n=120)** | | | **French cohort (n=54)** | | |
| --- | --- | --- | --- | --- | --- | --- | --- |
| **Number of sites*** | | **18 (100)** | | | **9 (100)** | | |
| **Number of countries*** | | **8 (100)** | | | **1 (100)** | | |
| Age | n | 120 (100) | | | 54 (100) | | |
|  | Median | 63.0 | | | 69.0 | | |
|  | Q1, Q3 | 56.5, 72.0 | | | 60.0, 73.0 | | |
|  | Min, Max | 29, 86 | | | 45.0, 88.0 | | |
| Sex | n | 120 (100) | | | 54 (100) | | |
|  | Female | 51 (42.5) | | | 22 (40.7) | | |
|  | Male | 65 (54.2) | | | 32 (59.3) | | |
|  | Unknown | 4 (3.3) | | | 0 (0) | | |
| Time since diagnosis (months) | n | 118 (98.3) | | | 54 (100) | | |
|  | Median | 6.6 | | | 5.2 | | |
|  | Q1, Q3 | 2.2, 15.4 | | | 2.2, 10.5 | | |
| ECOG status | n | 117 (97.5) | | | 53 (98.1) | | |
|  | 0-Fully Active | 69 (57.5) | | | 28 (51.9) | | |
|  | 1-Restricted | 39 (32.5) | | | 22 (40.7) | | |
|  | 2 or higher | 9 (7.5) | | | 3 (5.6) | | |
| Extra-hepatic disease prior to treatment | n | 120 | | | 54 (100) | | |
|  | No | 85 (70.8) | | | 39 (72.2) | | |
|  | Yes | 35 (29.2) | | | 15 (27.8) | | |
| Ascites | n | 120 (100) | | | 54 (100) | | |
|  | No | 108 (90) | | | 53 (98.1) | | |
|  | Yes | 12 (10) | | | 1 (1.9) | | |
| Cirrhosis | n | 120 (100) | | | 54 (100) | | |
|  | No | 105 (87.5%) | | | 48 (88.9) | | |
|  | Yes | 15 (12.5%) | | | 6 (11.1) | | |
| Location of liver tumours | n | 120 (100) | | | 54 (100) | | |
|  | Bilobar | 68 (56.7) | | | 18 (33.3) | | |
|  | Left only | 17 (14.2) | | | 10 (18.5) | | |
|  | Right only | 35 (29.2) | | | 26 (48.1) | | |
| Number of liver tumours | n | 120 (100) | | | 54 (100) | | |
|  | 1 | 44 (36.7) | | | 36 (66.7) | | |
|  | 2-5 | 25 (20.8) | | | 10 (18.5) | | |
|  | 6-9 | 10 (8.3) | | | 1 (1.9) | | |
|  | 10 or more | 10 (8.3) | | | 2 (3.7) | | |
|  | Uncountable | 31 (25.8) | | | 5 (9.3) | | |
| Methodology for determining the dose | n | 120 (100) | | | 54 (100) | | |
|  | BSA or modified BSA | 79 (65.8) | | | 8 (14.8) | | |
|  | Partition model | 41 (34.2) | | | 46 (85.2) | | |
|  | | **Whole liver** | **Right lobe** | **Left lobe** | **Whole liver** | **Right lobe** | **Left lobe** |
| Percentage of tumour invasion in the liver | n | 77 (64.2) | 35 (29.2) | 37 (30.8) | 46 | 8 | 8 |
|  | <10% | 27 (22.5) | 15 (12.5) | 16 (13.3) | 19 (35.2) | 1 (1.9) | 6 (11.1) |
|  | 10-20% | 27 (22.5) | 10 (8.3) | 9 (7.5) | 10 (18.5) | 5 (9.3) | 1 (1.9) |
|  | >20% | 23 (19.2) | 10 (8.3) | 12 (10) | 17 (31.5) | 2 (3.7) | 1 (1.9) |
| Prescribed activity (Giga-becquerel) | n | 49 | 71 | 71 | 42 | 12 | 12 |
|  | Median | 1.3 | 1.1 | 0.7 | 1 | 0.4 | 1.2 |
|  | Q1, Q3 | 1.0, 1.7 | 0.6, 1.4 | 0.0, 1.00 | 0.7, 1.4 | 0, 0.7 | 1, 1.4 |
| Liver treatment target | n | 120 (100) | | | 54 (100) | | |
|  | Whole liver (single catheter) | 13 (10.8) | | | 0 (0) | | |
|  | Whole liver (split administration, single session) | 26 (21.7) | | | 6 (11.1) | | |
|  | Whole liver (sequential lobar, two sessions) | 17 (14.2) | | | 0 (0) | | |
|  | Right lobe | 33 (27.5) | | | 29 (53.7) | | |
|  | Left lobe | 20 (16.7) | | | 14 (25.9) | | |
|  | Segmental | 11 (9.2) | | | 5 (9.3) | | |
| Delivered activity within 90% of prescribed (technical success) | n | 120 | | | 54 | | |
|  | No | 3 (2.5) | | | 1 (1.9) | | |
|  | Yes | 117 (97.5) | | | 53 (98.1) | | |
| Albumin (g/dL) | n | 101 (84.2) | | | 40 (74) | | |
|  | Median (IQR) | 3.9 (3.4, 4.2) | | | 3.7 (3.3, 3.9) | | |
| Bilirubin (µmol/L) | n | 120 (100) | | | 52 (96.3) | | |
|  | Median (IQR) | 10.0 (7.0, 14.6) | | | 10.0 (8.0, 14.0) | | |
| ALBI score | n | 101 (84.2) | | | 39 (72.2) | | |
|  | A1 | 57 (47.5) | | | 15 (27.8) | | |
|  | A2 | 43 (35.8) | | | 24 (44.4) | | |
|  | A3 | 1 (0.8) | | | 0 (0) | | |
| INR | n | 90 (75) | | | 44 (81.5) | | |
|  | Median (IQR) | 1.1 (1.0, 1.1) | | | 1.0 (1.0, 1.1) | | |
|  | ≤1 | 33 (27.5) | | | 18 (33.3) | | |
|  | >1 | 57 (47.5) | | | 26 (48.1) | | |
| N (%)  ALBI: Albumin-Bilirubin; BSA: Body Surface Area; ECOG: Eastern Cooperative Oncology Group; ICC: intrahepatic cholangiocarcinoma; INR: International Normalised Ratio; IQR: Interquartile Range.  *One site that was enrolled in CIRT before CIRT-FR was initiated switched from CIRT to CIRT-FR in August 2017. Therefore, the total number of sites and countries adds up to 26 and 8, respectively.  Categories where percentages (%) do not add up to 100 are due to missing information. | | | | | | | |

**Treatments before and after TARE**

| **Category** | **Subcategory** | **European cohort (n=120)** | **French cohort (n=54)** |
| --- | --- | --- | --- |
| Intention of treatment ^a^ | n | 120 (100) | 54 (100) |
|  | Palliative | 83 (69.2) | 45 (83.3) |
|  | Down-sizing / downstaging | 25 (20.8) | 8 (14.8) |
|  | Bridge to ablation | 7 (5.8) | 1 (1.9) |
|  | Bridge to liver surgery | 3 (2.5) | 0 (0) |
|  | Bridge to liver transplant | 2 (1.7) | 0 (0) |
| Hepatic procedures prior to TARE | n | 120 (100) | 54 (100) |
|  | Yes | 41 (34.2) | 8 (14.8) |
|  | No | 79 (65.8) | 46 (85.2) |
| Type of hepatic procedures ^b^ | n | 41 (100) | 8 (100) |
|  | Surgical (any) | 32 (78.0) | 5 (62.5) |
|  | Ablation (any) | 7 (17.1) | 1 (12.5) |
|  | TACE (any) | 2 (4.9) | 0 (0) |
|  | Other embolotherapies (any) | 1 (2.4) | 0 (0) |
|  | Abdominal radiotherapy (any) | 5 (12.2) | 4 (50.0) |
| Systemic therapy prior to TARE | n | 120 (100) | 54 (100) |
|  | Yes | 73 (60.8) | 27 (50.0) |
|  | No | 47 (39.2) | 27 (50.0) |
| Number of systemic therapy lines | n | 72 (98.6) | 27 (100) |
|  | 1 Line | 41 (56.2) | 23 (85.2) |
|  | 2-5 Lines | 26 (35.6) | 4 (14.8) |
|  | 6 or more lines | 5 (6.9) | 0 (0) |
| Systemic therapy after TARE | n | 120 (100) | 54 (100) |
|  | Yes | 45 (37.5) | 39 (72.2) |
|  | No ^d^ | 72 (62.5) | 15 (27.8) |
| Number of systemic therapy lines | n | 44 (97.8) | 39 (100) |
|  | 1 Line | 17 (31.5) | 20 (51.3) |
|  | 2-5 Lines | 13 (28.9) | 18 (46.2) |
|  | 6 or more lines | 14 (31.1) | 1 (2.6) |
| Hepatic procedures after TARE | Yes | 20 (16.7) | 9 (16.7) |
|  | No ^d^ | 100 (83.3) | 43 (79.6) |
| Type of hepatic procedures after TARE ^b^ | n | 20 | 9 |
|  | Surgical (any) | 4 (20.0) | 8 (88.9) |
|  | Ablation (any) | 4 (20.0) | 1 (11.1) |
|  | TACE (any) | 1 (5.0) | 0 (0) |
|  | Other embolotherapies (any) | 2 (10.0) | 2 (22.2) |
|  | Abdominal radiotherapy (any) | 6 (30.0) | 6 (66.7) |
|  | Not reported | 3 (15.0) | 0 (0) |
| N (%)  ^a^ Intention of TARE is for first treatment.  ^b^ Patients can have multiple prior and post-TARE hepatic procedures.  ^c^ Patients can have multiple prior and post-TARE systemic therapies.  ^d^ No systemic therapy after TARE includes patients that were lost to follow-up or deceased before the first follow-up could be included (12 [10] for CIRT and 2 [3.7] for CIRT-FR).  Abbreviations: ICC: Intrahepatic Cholangiocarcinoma; TACE: Transarterial Chemoembolization; TARE: Transarterial Radioembolization.  Categories where percentages (%) do not add up to 100 are due to missing information. | | | |

## **Supplement 3: A baseline comparison between patients receiving first-line TARE versus first-line TARE plus concomitant systemic therapy**

| **Variable** | **Type** | **First line TARE** | **First-line TARE + concomitant systemic treatment** | **All** | **P-value** |
| --- | --- | --- | --- | --- | --- |
| Age (year) | n | 62 | 20 | 82 | 0.536 |
|  | Mean ± SD | 64.7 ± 12.2 | 66.6 ± 10.6 | 65.2 ± 11.8 |  |
|  | (Min;Max) | (33.0 ; 86.0) | (45.0 ; 88.0) | (33.0 ; 88.0) |  |
|  | Median [IQR] | 64.5 [57.0;75.0] | 66.5 [62.5;73.0] | 65.0 [58.0;73.0] |  |
| Gender | Female | 23 (37.1%) | 11 (55.0%) | 34 (41.5%) | 0.3948 |
|  | Male | 38 (61.3%) | 9 (45.0%) | 47 (57.3%) |  |
|  | Unknown | 1 (1.6%) | 0 (0.0%) | 1 (1.2%) |  |
| ECOG | 0 | 35 (56.5%) | 13 (65.0%) | 48 (58.5%) | 0.4697 |
|  | 1 | 21 (33.9%) | 7 (35.0%) | 28 (34.1%) |  |
|  | 2+3+4 | 6 (9.7%) | 0 (0.0%) | 6 (7.3%) |  |
| Extra-hepatic disease prior to treatment | No | 48 (77.4%) | 17 (85.0%) | 65 (79.3%) | 0.5449 |
|  | Yes | 14 (22.6%) | 3 (15.0%) | 17 (20.7%) |  |
| Ascites | No | 58 (93.5%) | 19 (95.0%) | 77 (93.9%) | 1 |
|  | Yes | 4 (6.5%) | 1 (5.0%) | 5 (6.1%) |  |
| Cirrhosis | No | 51 (82.3%) | 18 (90.0%) | 69 (84.1%) | 0.5049 |
|  | Yes | 11 (17.7%) | 2 (10.0%) | 13 (15.9%) |  |
| Location of liver tumors | Bilobar | 25 (40.3%) | 9 (45.0%) | 34 (41.5%) | 0.6615 |
|  | Left | 12 (19.4%) | 2 (10.0%) | 14 (17.1%) |  |
|  | Right | 25 (40.3%) | 9 (45.0%) | 34 (41.5%) |  |
| Tumour burden (nodules) | 1 | 36 (58.1%) | 13 (65.0%) | 49 (59.8%) | 0.7319 |
|  | 2-5 | 9 (14.5%) | 1 (5.0%) | 10 (12.2%) |  |
|  | >5 | 6 (9.7%) | 2 (10.0%) | 8 (9.8%) |  |
|  | Uncountable | 11 (17.7%) | 4 (20.0%) | 15 (18.3%) |  |
| Dose methodology | Partition model | 35 (56.5%) | 13 (65.0%) | 48 (58.5%) | 0.4998 |
|  | BSA/mBSA | 27 (43.5%) | 7 (35.0%) | 34 (41.5%) |  |
| Albumin (g/dL) | n | 55 | 14 | 69 | 0.3583 |
|  | Mean ± SD | 3.7 ± 0.5 | 3.9 ± 0.5 | 3.7 ± 0.5 |  |
|  | (Min;Max) | (2.8 ; 4.8) | (3.1 ; 4.6) | (2.8 ; 4.8) |  |
|  | Median [IQR] | 3.7 [3.4;4.2] | 3.8 [3.5;4.3] | 3.7 [3.4;4.2] |  |
| Bilirubin (µmol/L) | n | 62 | 19 | 81 | 0.7854 |
|  | Mean ± SD | 13.8 ± 9.2 | 17.6 ± 21.4 | 14.7 ± 13.1 |  |
|  | (Min;Max) : | (3.2 ; 48.2) | (4.6 ; 84.0) | (3.2 ; 84.0) |  |
|  | Median [IQR] | 10.4 [7.8;17.6] | 10.0 [8.0;14.0] | 10.0 [8.0;15.4] |  |
| Albumin-bilirubin (ALBI) score | n | 55 | 13 | 68 | 0.7561 |
|  | Mean ± SD | -2.5 ± 0.5 | -2.6 ± 0.5 | -2.5 ± 0.5 |  |
|  | (Min;Max) | (-3.6 ; -1.5) | (-3.4 ; -1.9) | (-3.6 ; -1.5) |  |
|  | Median [IQR] | -2.5 [-3.0;-2.1] | -2.6 [-2.8;-2.1] | -2.5 [-2.9;-2.1] |  |
| Albumin-bilirubin (ALBI) grade | 1 | 22 (40.0%) | 7 (53.8%) | 29 (42.6%) | 0.3640 |
|  | 2 | 33 (60.0%) | 6 (46.2%) | 39 (57.4%) |  |
| International Normalised Ratio (INR) | n | 53 | 13 | 66 | 0.0175 |
|  | Mean ± SD | 1.1 ± 0.2 | 1.0 ± 0.1 | 1.1 ± 0.2 |  |
|  | (Min;Max) | (0.9 ; 2.4) | (0.9 ; 1.2) | (0.9 ; 2.4) |  |
|  | Median [IQR] | 1.1 [1.0;1.2] | 1.0 [1.0;1.0] | 1.0 [1.0;1.1] |  |
| International Normalised Ratio (INR) | <=1 | 16 (30.2%) | 8 (61.5%) | 24 (36.4%) | 0.0532 |
|  | >1 | 37 (69.8%) | 5 (38.5%) | 42 (63.6%) |  |

## **Supplement 4: Time from diagnosis to TARE treatment per treatment line**

| **Type** | **First line TARE** | **First line TARE + CT** | **Second line TARE** | **>2nd line TARE** | **All** |
| --- | --- | --- | --- | --- | --- |
| n | 62 | 20 | 53 | 21 | 156 |
| Mean ± SD | 3.8 ± 4.8 | 2.8 ± 2.8 | 15.3 ± 19.1 | 25.9 ± 17.8 | 10.6 ± 15.4 |
| (Min;Max) | (0.1 ; 27.3) | (0.7 ; 13.2) | (3.2 ; 135.6) | (4.0 ; 72.1) | (0.1 ; 135.6) |
| Median [IQR] | 2.1 [1.2;4.3] | 2.2 [1.0;3.3] | 10.2 [7.6;17.3] | 21.4 [13.9;27.9] | 5.5 [2.1;13.4] |

## **Supplement 5: Univariable analysis for overall survival.**

Cox model, p-value 0.05.

| **Variable** | **Threshold** | **Median (95% CI)** | **p value** | **HR (95% CI)** | **p value HR** |
| --- | --- | --- | --- | --- | --- |
| Gender | Female | 16.6 (10.9-22.1) | 0.5471 | 0.89 (0.61-1.30) | 0.5473 |
|  | Male | 13.7 (9.3-19.5) | . |  | . |
| Position of TARE in the continuum of care | 1^st^ line TARE | 16.2 (9.0-27.2) | 0.0028 |  |  |
|  | 1^st^ line TARE plus CT | 32.5 (11.8-27.0) | . | 0.64 (0.34-1.22) | 0.1730 |
|  | 2^nd^ line TARE | 12.0 (8.2-20.8) |  | 1.28 (0.82-2.02) | 0.2817 |
|  | >2^nd^ line TARE | 9.3 (4.5-14.7) | . | 2.37 (1.31-4.29) | 0.0042 |
| ECOG | 0 | 17.1 (13.7-22.1) | 0.1598 |  | . |
|  | 1 | 9.3 (7.0-19.5) | . | 1.39 (0.93-2.06) | 0.1076 |
|  | 2+3+4 | 10.0 (3.5-.) | . | 1.63 (0.81-3.29) | 0.1749 |
| Extra-hepatic disease prior to treatment | No | 18.4 (11.8-22.9) | 0.0023 | 0.55 (0.37-0.81) | 0.0026 |
|  | Yes | 10.9 (6.2-14.7) | . |  | . |
| Location of liver tumours | Bilobar | 11.8 (9.3-15.4) | 0.0929 | 1.48 (0.84-2.60) | 0.1707 |
|  | Left | 21.9 (10.5-36.8) | . |  | . |
|  | Right | 21.4 (10.1-32.5) | . | 0.98 (0.53-1.79) | 0.9368 |
| Ascites | No | 15.4 (11.8-20.6) | 0.0950 | 0.56 (0.28-1.12) | 0.1000 |
|  | Yes | 7.7 (2.1-27.7) | . |  | . |
| Cirrhosis | No | 16.6 (13.2-21.1) | 0.0051 | 0.47 (0.28-0.81) | 0.0062 |
|  | Yes | 7.7 (3.8-10.1) | . |  | . |
| Prior surgery | No | 14.0 (9.7-20.6) | 0.8328 | 1.05 (0.66-1.68) | 0.8307 |
|  | Yes | 14.7 (10.8-21.4) | . |  | . |
| Dose methodology | BSA/mBSA | 12.0 (9.4-16.6) | 0.0363 |  | . |
|  | Partition model | 17.1 (10.1-26.2) | . | 0.67 (0.46-0.98) | 0.0379 |
| Treatment intention | Ablation, bridge to surgery, transplant | 17.9 (3.0-.) | 0.6705 |  | . |
|  | Down-sizing/down-staging | 12.0 (7.7-18.4) | . | 1.21 (0.51-2.86) | 0.6662 |
|  | Palliative | 15.3 (11.4-21.1) | . | 0.97 (0.45-2.10) | 0.9340 |
| Prior locoregional treatments | No | 15.3 (9.5-20.8) | 0.8299 | 1.05 (0.69-1.59) | 0.8324 |
|  | Yes | 14.7 (11.4-21.1) | . |  | . |
| Locoregional treatments after TARE | No | 11.8 (9.7-16.6) | 0.0101 | 1.98 (1.17-3.37) | 0.0116 |
|  | Yes | 27.7 (16.2-37.0) | . |  | . |
| Systemic therapy after TARE | No | 9.3 (7.0-14.0) | 0.0008 | 1.87 (1.29-2.71) | 0.0010 |
|  | Yes | 20.6 (14.7-26.2) | . |  | . |
| Additional treatments after TARE | No | 8.2 (5.7-11.2) | 0.0000 | 2.36 (1.62-3.43) | 0.0000 |
|  | Yes | 20.8 (15.4-27.7) | . |  | . |
| Time from diagnosis to treatment (months) | <6.1 | 17.1 (10.8-26.2) | 0.1086 |  | . |
|  | >=6.1 | 13.7 (9.7-16.6) | . | 1.36 (0.93-1.97) | 0.1100 |
| Total tumour to liver (%) | <10% | 20.8 (10.8-31.6) | 0.3656 |  | . |
|  | 10-20% | 16.6 (10.9-.) | . | 0.90 (0.50-1.60) | 0.7104 |
|  | >20% | 8.2 (5.3-22.1) | . | 1.33 (0.79-2.22) | 0.2827 |
| Right tumour to liver (%) | <10% | 10.9 (7.8-14.7) | 0.4136 |  | . |
|  | 10-20% | 17.1 (6.1-.) | . | 0.59 (0.25-1.37) | 0.2158 |
|  | >20% | 15.4 (2.6-27.7) | . | 0.67 (0.29-1.58) | 0.3647 |
| Left tumour to liver (%) | <10% | 17.1 (9.3-27.7) | 0.2248 |  | . |
|  | 10-20% | 11.2 (4.7-.) | . | 1.18 (0.42-3.31) | 0.7605 |
|  | >20% | 9.4 (5.0-11.8) | . | 1.95 (0.89-4.27) | 0.0942 |
| ALBI grade | 1 | 16.6 (13.7-21.1) | 0.7977 |  | . |
|  | 2 | 10.9 (7.8-17.9) | . | 1.07 (0.71-1.62) | 0.7331 |
|  | 3 | 11.8 (.-.) | . | 1.83 (0.25-13.38) | 0.5495 |
| INR | <=1 | 19.1 (15.3-26.2) | 0.0967 |  | . |
|  | >1 | 10.8 (8.7-15.4) | . | 1.43 (0.94-2.19) | 0.0982 |
| Levels of significance: p <0.05 (Log-rank test [Mantel-Haenszel version]). Abbreviations: ALBI: Albumin-Bilirubin; BSA: Body Surface Area; CI: Confidence Interval; ECOG: Eastern Cooperative Oncology Group; ICC: intrahepatic cholangiocarcinoma; HR: Hazard Ratio; TARE: Transarterial Radioembolization. | | | | | |

## **Supplement 6: Univariable analysis for progression-free survival.**

Cox model, p-value 0.05.

| **Variable** | **Threshold** | **Median (95% CI)** | **p value** | **HR (95% CI)** | **p value HR** |
| --- | --- | --- | --- | --- | --- |
| Gender | Female | 6.3 (4.3-8.5) | 0.3300 | 1.18 (0.85-1.64) | 0.3315 |
|  | Male | 5.3 (3.9-7.0) | . |  | . |
| Position of TARE in the continuum of care | 1^st^ line TARE | 7.4 (3.9-11.0) | 0.0012 |  |  |
|  | 1^st^ line TARE plus CT | 11.3 (6.1-14.0) |  | 0.80 (0.46-1.40) | 0.4405 |
|  | 2^nd^ line TARE | 5.1 (3.1-6.4) | . | 1.56 (1.04-2.33) | 0.0300 |
|  | >2^nd^ line TARE | 3.5 (2.5-4.3) | . | 2.36 (1.39-4.04) | 0.0016 |
| ECOG | 0 | 7.1 (5.0-9.7) | 0.2052 |  | . |
|  | 1 | 5.5 (3.8-7.0) | . | 1.29 (0.91-1.83) | 0.1559 |
|  | 2+3+4 | 4.0 (2.0-10.5) | . | 1.55 (0.82-2.92) | 0.1766 |
| Extra-hepatic disease prior to treatment | No | 6.9 (5.2-8.6) | 0.0025 | 0.59 (0.42-0.83) | 0.0028 |
|  | Yes | 3.6 (3.0-6.0) | . |  | . |
| Location of liver tumours | Bilobar | 5.3 (4.1-6.9) | 0.0665 | 1.00 (0.64-1.58) | 0.9897 |
|  | Left | 6.3 (3.0-9.6) | . |  | . |
|  | Right | 7.1 (3.7-11.8) | . | 0.66 (0.40-1.08) | 0.0986 |
| Ascites | No | 6.1 (4.4-7.4) | 0.0535 | 0.55 (0.29-1.02) | 0.0574 |
|  | Yes | 2.4 (1.6-8.7) | . |  | . |
| Cirrhosis | No | 6.2 (4.9-8.2) | 0.3232 | 0.77 (0.47-1.29) | 0.3227 |
|  | Yes | 3.8 (2.4-6.1) | . |  | . |
| Prior surgery | No | 6.3 (5.0-8.2) | 0.3929 | 0.84 (0.57-1.25) | 0.3918 |
|  | Yes | 4.1 (3.1-6.9) | . |  | . |
| Dose methodology | BSA/mBSA | 5.3 (3.8-8.2) | 0.5612 |  | . |
|  | Partition model | 6.1 (4.9-7.4) | . | 0.91 (0.66-1.26) | 0.5651 |
| Treatment intention | Ablation, bridge to surgery, transplant | 10.7 (1.9-12.7) | 0.7153 |  | . |
|  | Down-sizing/down-staging | 8.2 (3.8-10.5) | . | 0.84 (0.41-1.70) | 0.6234 |
|  | Palliative | 5.6 (4.1-6.4) | . | 1.00 (0.54-1.87) | 0.9948 |
| Prior locoregional treatments | No | 6.3 (5.2-8.3) | 0.3411 | 0.84 (0.59-1.20) | 0.3399 |
|  | Yes | 4.1 (3.2-6.2) | . |  | . |
| Locoregional treatments after TARE | No | 5.3 (4.0-6.3) | 0.0893 | 1.47 (0.94-2.29) | 0.0915 |
|  | Yes | 9.0 (4.3-11.7) | . |  | . |
| Systemic therapy after TARE | No | 4.4 (3.8-6.2) | 0.7774 | 1.05 (0.76-1.45) | 0.7765 |
|  | Yes | 6.8 (5.3-8.6) | . |  | . |
| Additional treatments after TARE | No | 4.3 (3.5-5.7) | 0.2500 | 1.21 (0.87-1.69) | 0.2489 |
|  | Yes | 7.2 (5.7-8.7) | . |  | . |
| Time from diagnosis to treatment (months) | <6.1 | 7.5 (5.3-10.2) | 0.0054 |  | . |
|  | >=6.1 | 4.4 (3.8-6.2) | . | 1.59 (1.15-2.22) | 0.0057 |
| Total tumour to liver (%) | <10% | 7.3 (4.4-12.0) | 0.0602 |  | . |
|  | 10-20% | 7.1 (4.4-9.3) | . | 1.44 (0.89-2.34) | 0.1414 |
|  | >20% | 4.4 (3.5-7.0) | . | 1.74 (1.09-2.80) | 0.0210 |
| Right tumour to liver (%) | <10% | 3.6 (2.9-9.0) | 0.5886 |  | . |
|  | 10-20% | 6.9 (1.6-22.1) | . | 0.66 (0.30-1.46) | 0.3034 |
|  | >20% | 5.0 (2.4-19.1) | . | 0.81 (0.37-1.77) | 0.5890 |
| Left tumour to liver (%) | <10% | 5.7 (3.1-22.1) | 0.1062 |  | . |
|  | 10-20% | 3.5 (1.0-6.9) | . | 2.22 (0.94-5.22) | 0.0689 |
|  | >20% | 3.9 (2.4-9.0) | . | 1.93 (0.90-4.13) | 0.0920 |
| ALBI grade | 1 | 6.2 (4.4-7.5) | 0.7030 |  | . |
|  | 2 | 3.9 (3.1-7.1) | . | 1.09 (0.76-1.56) | 0.6555 |
|  | 3 | 3.9 (.-.) | . | 2.12 (0.29-15.45) | 0.4571 |
| INR | <=1 | 6.9 (5.2-8.6) | 0.5332 |  | . |
|  | >1 | 4.0 (3.3-6.0) | . | 1.13 (0.77-1.64) | 0.5313 |
| Levels of significance: p <0.05 (Log-rank test [Mantel-Haenszel version]). Abbreviations: ALBI: Albumin-Bilirubin; BSA: Body Surface Area; CI: Confidence Interval; ECOG: Eastern Cooperative Oncology Group; ICC: intrahepatic cholangiocarcinoma; HR: Hazard Ratio; TARE: Transarterial Radioembolization. | | | | | |

## **Supplement 7: Univariable analysis for hepatic progression-free survival.**

Cox model, p-value 0.05.

| **Variable** | **Threshold** | **Median (95% CI)** | **p value** | **HR (95% CI)** | **p value HR** |
| --- | --- | --- | --- | --- | --- |
| Gender | Female | 7.2 (5.7-9.0) | 0.7571 | 1.05 (0.76-1.46) | 0.7594 |
|  | Male | 5.8 (4.1-8.2) | . |  | . |
| Position of TARE in the continuum of care | 1^st^ line TARE | 7.5 (4.3-11.0) | 0.0005 |  |  |
|  | 1^st^ line TARE plus CT | 13.8 (8.3-28.1) |  | 0.63 (0.35-1.12) | 0.1140 |
|  | 2^nd^ line TARE | 5.7 (3.8-7.2) | . | 1.54 (1.03-2.30) | 0.0337 |
|  | >2^nd^ line TARE | 3.9 (2.6-5.7) | . | 2.15 (1.27-3.67) | 0.0047 |
| ECOG | 0 | 7.4 (5.8-10.2) | 0.5079 |  | . |
|  | 1 | 5.7 (3.9-8.1) | . | 1.23 (0.86-1.74) | 0.2582 |
|  | 2+3+4 | 7.1 (3.4-11.8) | . | 1.19 (0.63-2.23) | 0.5988 |
| Extra-hepatic disease prior to treatment | No | 7.4 (5.7-9.3) | 0.0032 | 0.60 (0.42-0.84) | 0.0035 |
|  | Yes | 5.0 (3.1-6.4) | . |  | . |
| Location of liver tumours | Bilobar | 6.0 (4.4-7.2) | 0.0586 | 1.06 (0.67-1.66) | 0.8155 |
|  | Left | 6.4 (3.0-9.6) | . |  | . |
|  | Right | 7.4 (4.1-12.7) | . | 0.68 (0.41-1.11) | 0.1246 |
| Ascites | No | 6.8 (5.6-8.2) | 0.0812 | 0.58 (0.31-1.08) | 0.0856 |
|  | Yes | 3.0 (2.0-9.5) | . |  | . |
| Cirrhosis | No | 7.1 (5.7-8.6) | 0.2458 | 0.74 (0.45-1.23) | 0.2448 |
|  | Yes | 3.9 (2.4-7.0) | . |  | . |
| Prior surgery | No | 7.2 (5.7-8.7) | 0.2045 | 0.78 (0.53-1.15) | 0.2035 |
|  | Yes | 4.1 (3.1-7.1) | . |  | . |
| Dose methodology | BSA/mBSA | 6.2 (4.0-9.0) | 0.3835 |  | . |
|  | Partition model | 6.6 (5.3-8.5) | . | 0.86 (0.62-1.20) | 0.3865 |
| Treatment intention | Ablation, bridge to surgery, transplant | 10.7 (2.1-12.7) | 0.8991 |  | . |
|  | Down-sizing/down-staging | 8.2 (4.1-10.5) | . | 0.89 (0.44-1.81) | 0.7550 |
|  | Palliative | 6.0 (4.4-7.4) | . | 0.99 (0.53-1.84) | 0.9696 |
| Prior locoregional treatments | No | 7.4 (5.7-8.7) | 0.2158 | 0.80 (0.56-1.14) | 0.2144 |
|  | Yes | 4.4 (3.2-6.9) | . |  | . |
| Locoregional treatments after TARE | No | 6.1 (4.4-7.2) | 0.1996 | 1.34 (0.86-2.09) | 0.2013 |
|  | Yes | 9.0 (4.3-11.7) | . |  | . |
| Systemic therapy after TARE | No | 5.7 (3.9-8.1) | 0.5146 | 1.12 (0.80-1.55) | 0.5129 |
|  | Yes | 7.2 (6.1-9.1) | . |  | . |
| Additional treatments after TARE | No | 5.0 (3.9-7.1) | 0.1735 | 1.26 (0.90-1.75) | 0.1732 |
|  | Yes | 7.4 (6.3-9.1) | . |  | . |
| Time from diagnosis to treatment (months) | <6.1 | 8.6 (6.1-10.7) | 0.0021 |  | . |
|  | >=6.1 | 5.7 (4.0-7.1) | . | 1.68 (1.20-2.35) | 0.0023 |
| Total tumour to liver (%) | <10% | 8.1 (5.7-12.0) | 0.1801 |  | . |
|  | 10-20% | 7.1 (4.4-9.3) | . | 1.49 (0.92-2.42) | 0.1052 |
|  | >20% | 5.3 (3.7-8.2) | . | 1.45 (0.90-2.32) | 0.1254 |
| Right tumour to liver (%) | <10% | 5.7 (3.1-9.5) | 0.7259 |  | . |
|  | 10-20% | 7.5 (1.6-22.1) | . | 0.73 (0.33-1.61) | 0.4376 |
|  | >20% | 5.0 (2.4-19.1) | . | 0.93 (0.42-2.05) | 0.8587 |
| Left tumour to liver (%) | <10% | 7.4 (3.1-22.1) | 0.0905 |  | . |
|  | 10-20% | 4.8 (1.0-9.3) | . | 2.33 (0.98-5.52) | 0.0555 |
|  | >20% | 5.0 (2.9-9.3) | . | 1.97 (0.91-4.25) | 0.0849 |
| ALBI grade | 1 | 6.3 (4.9-8.2) | 0.6507 |  | . |
|  | 2 | 6.3 (3.8-9.3) | . | 0.97 (0.67-1.39) | 0.8597 |
|  | 3 | 3.9 (.-.) | . | 2.41 (0.33-17.63) | 0.3849 |
| INR | <=1 | 6.9 (5.2-8.6) | 0.9486 |  | . |
|  | >1 | 5.7 (3.9-7.4) | . | 1.01 (0.70-1.48) | 0.9472 |
| Levels of significance: p <0.05 (Log-rank test [Mantel-Haenszel version]). Abbreviations: ALBI: Albumin-Bilirubin; BSA: Body Surface Area; CI: Confidence Interval; ECOG: Eastern Cooperative Oncology Group; ICC: intrahepatic cholangiocarcinoma; HR: Hazard Ratio; TARE: Transarterial Radioembolization. | | | | | |

## **Supplement 8: Safety data from the European and French cohorts**

**Safety European cohort (n=120)**

| **Category** | **Subcategory** | **All grades** | **Grade 3-5** |
| --- | --- | --- | --- |
| Patients with adverse events | No. of patients with at least one adverse event | 49 (40.8%) | 13 (11.7%) |
| Adverse events (all) | Abdominal pain | 25 (20.8%) | 4 (3.3%) |
|  | Fatigue | 23 (19.2%) | 2 (1.7%) |
|  | Fever | 7 (5.8%) | 0 (0%) |
|  | Nausea | 14 (11.7%) | 0 (0%) |
|  | Vomiting | 9 (7.5%) | 0 (0%) |
|  | Gastrointestinal ulceration | 5 (4.2%) | 0 (0%) |
|  | Gastritis | 2 (1.7%) | 1 (0.8%) |
|  | Radiation cholecystitis | 1 (0.8%) | 1 (0.8%) |
|  | Radiation pancreatitis |  | 1 (0.8%) |
|  | Radioembolization-Induced Liver Disease | 3 (2.5%) | 2 (1.7%) |
|  | Other | 25 (20.8%) | 8 (6.5%) |

**Safety French cohort (n=54)**

| **Category** | **Subcategory** | **All grades** | **Grade 3-5** |
| --- | --- | --- | --- |
| Patients with adverse events | No. of patients with at least one adverse event | 40 (74.1%) | 15 (27.8%) |
| Adverse events (all) | Abdominal pain | 9 (16.7%) | 1 (1.9%) |
|  | Fatigue | 30 (55.6%) | 3 (5.6%) |
|  | Fever | 2 (3.7%) | 0 (0%) |
|  | Nausea | 17 (31.5%) | 0 (0%) |
|  | Vomiting | 4 (7.4%) | 0 (0%) |
|  | Gastrointestinal ulceration | 1 (1.9%) | 1 (1.9%) |
|  | Gastritis | 0 (0%) | 0 (0%) |
|  | Radiation cholecystitis | 0 (0%) | 0 (0%) |
|  | Radiation pancreatitis | 0 (0%) | 0 (0%) |
|  | Radioembolization-Induced Liver Disease | 1 (1.9%) | 1 (1.9%) |
|  | Other | 34 (63%) | 14 (25.9%) |
